# Acute *Toxoplasma gondii* Infection Drives Gut Microbiome Dysbiosis and Functional Disruption in Mice as Revealed by Metagenomic Sequencing

**DOI:** 10.3390/microorganisms13092056

**Published:** 2025-09-04

**Authors:** Yidan Wang, Caiqin Deng, Minmin Sui, Penghao Wei, Bofang Duan, Zhao Li, Fengcai Zou

**Affiliations:** 1 Faculty of Animal Science and Technology, Yunnan Agricultural University, Kunming 650201, China; wangyidan318@163.com (Y.W.); 15666251052@163.com (M.S.); weipenghao0414@163.com (P.W.); 2 The Yunnan Key Laboratory of Veterinary Etiological Biology, College of Veterinary Medicine, Yunnan Agricultural University, Kunming 650201, China; dengcaiqin1998@163.com (C.D.); duanbofang@126.com (B.D.); 3 Animal Research and Resource Center, School of Life Sciences, Yunnan University, Kunming 650201, China; 4 Yunnan Province Center for Animal Disease Control and Prevention, Kunming 650201, China

**Keywords:** *Toxoplasma gondii*, gut microbiome, metagenomic sequencing, dysbiosis, KEEG and CAZy analyses

## Abstract

*Toxoplasma gondii* is a widely distributed intracellular parasite that disrupts host immune and metabolic homeostasis. Although accumulating evidence highlights the role of gut microbiota in parasitic infections, the effects of acute *T. gondii* infection on host gut microbial ecology remain poorly understood. In this study, metagenomic sequencing technology was used to systematically analyze the composition and functional alterations of the ileal microbiota in BALB/c mice on day 10 post-infection. Compared to uninfected controls, *T*. *gondii* infected mice exhibited a significant reduction in microbial diversity and a pronounced shift in community structure. Notably, there was an expansion of Proteobacteria, particularly the Enterobacteriaceae family, alongside a marked decline in beneficial taxa such as Actinobacteria and Bacillota. Functional annotation using the KEGG and CAZy databases revealed enrichment of metabolic pathways related to glycolysis/gluconeogenesis, O-antigen nucleotide sugar biosynthesis, bacterial secretion systems, and biofilm formation-*Escherichia coli* in the infected microbiota. These findings provide novel insights into the dysbiosis of gut microbiota and host-microbe interactions during acute *T. gondii* infection.

## 1. Introduction

*T. gondii* is an obligate intracellular parasite that is widely distributed globally and infects approximately one-third of humans and a wide range of warm-blooded animals [1,2]. Hosts with a robust immune function often exhibit no symptoms or only mild symptoms after infection. However, in individuals with weakened immunity and congenital infections, acute toxoplasmosis can lead to severe and potentially life-threatening pathological outcomes [3].

*T. gondii* is primarily transmitted through the oral route. Upon the ingests the cysts or oocysts, the parasites invade the epithelial cells of the small intestine and spread to various tissues and organs throughout the body to reproduce asexually [4]. Traditionally, the pathogenic mechanism of *T. gondii* infection has been mainly attributed to the host immune response and the manipulation of host cell signaling pathways by the parasite [5,6]. It is worth noting that the intestine is not only the gateway for *T. gondii* to invade, but also harbors a complex gut microbiota that may play a potential key regulatory role in the infection process [7]. Recent studies have demonstrated that under normal physiological conditions, *T. gondii* infection can activate the synergistic defense mechanisms of the mucosal immune system and the gut microbiota. However, microbial disruption may exacerbate disease severity by synergistically promoting gut inflammation in conjunction with *T. gondii* [4]. These findings suggest the existence of a dynamic and complex interaction network among *T. gondii*, the host mucosal immune system, and the gut microbiota during infection.

The gut microbiota constitutes a highly complex and dynamic ecosystem that plays a critical role in host nutrient metabolism, immune homeostasis, and maintenance of the gut barrier function [8]. Dysbiosis of the gut flora has been closely associated with the onset and progression of various diseases, including infections, inflammatory conditions, and metabolic disorders [9]. While interactions between bacterial or viral infections and the gut microbiota have been extensively studied, the effects of protozoan parasites, particularly *T. gondii*, on gut microbial composition and function remain largely underexplored. Previous studies have shown that parasitic infections can significantly alter the gut microbiota, leading to reduced species diversity and abnormal proliferation of opportunistic pathogens [10,11]. However, the comprehensive effects of acute *T. gondii* infection on microbial taxonomic structure, functional capacity, and microbiota-mediated pathogenic mechanisms remain poorly understood.

To address this gap, the present study employed metagenomic sequencing to comprehensively characterize the ileal microbiota of mice during acute *T. gondii* infection. High-resolution taxonomic profiling was conducted alongside in-depth functional annotation using the KEGG and CAZy databases. By integrating taxonomic and functional analyses, this study aims to elucidate the mechanisms by which *T. gondii* disrupts gut microbial ecology and identify potential functional pathways involved in infection-induced dysbiosis. These findings will provide new theoretical insights and experimental evidence to advance our understanding of the complex interplay among the gut microbiota, *T. gondii*, and host immune responses.

## 2. Materials and Methods

### 2.1. Experimental Design and Sample Collection

Eight-week-old female BALB/c mice were purchased from Yunnan University. All mice were housed in individually ventilated cages and had ad libitum access to food and water. The ME49 strain of *T. gondii* (Type II), used for constructing infectious models, was obtained from Yunnan University. After 1 week of acclimatization, the mice were randomly divided into two groups (*n* = 3 per group): an acute infection (BCA) group and a control group for the acute phase (BCN). The mice in the infected groups were orally infected with 100 *T. gondii* cysts, whereas those in the control groups received 0.2 mL of sterile phosphate-buffered solution. On day 10 post-infection (acute phase; BCA), the mice were euthanized, and at least 2 g of ileal content was aseptically collected and stored at −80 °C for subsequent DNA extraction. The Yunnan University Ethics Committee approved all animal experiments (Approval No. YNU20241024; date: 2 February 2024).

### 2.2. DNA Extraction, Metagenomic Library Preparation, and Sequencing

DNA was extracted from samples using the MagPure Stool DNA KF Kit B (MAGEN, Guangzhou, China). For metagenomic library preparation, the BGI Optimal DNA Library Prep Kit (BGI, Shenzhen, China) was employed. Briefly, the extracted DNA underwent fragmentation and size selection via magnetic bead purification, followed by end repair and adenylation. Subsequently, sequencing adapters were ligated to the fragments, followed by PCR amplification and quality control of the library. After library quality assessment, the double-stranded DNA libraries were denatured into single strands and circularized. Linear DNA remnants were then removed via enzymatic digestion, and the circular single-stranded DNA templates were amplified using rolling circle amplification (RCA) with phi29 polymerase to produce DNA nanoballs (DNBs).

### 2.3. Bioinformatics Analysis of Metagenomic Sequencing Data

All the raw data were trimmed by SOAPnuke v.1.5.2 [12]. The trimmed reads were mapped to the host genome using SOAP2 v.2.21 [13] software to identify and remove host-originated reads (only for samples of host origin). High-quality reads were then subjected to de novo assembly using MEGAHIT v.1.2.9 software [14]. Contigs shorter than 200 base pairs were excluded from further analysis. Genes were predicted over contigs using MetaGeneMark v.3.38 [15], followed by removal of redundant gene sequences via CD-HIT v.4.8.1 [16] at a sequence identity cutoff of 95% and coverage cutoff of 90%. To construct the gene abundance matrix, the Salmon v.1.6.0 software [17] is used for quantification. For functional annotation, predicted gene protein sequences were aligned against functional databases (e.g., KEGG, CAZy) using DIAMOND v.2.1.8 [18] with an E-value cutoff of 1 × 10^−5^. Taxonomic annotations were assigned based on the Kraken v.2 LCA algorithm [19].

### 2.4. Differential Abundance Analysis and Statistical Assessment of Diversity Across Groups

Species annotations were obtained from the NR database, and species abundance was determined based on the relative expression levels of corresponding genes. The α diversity was quantified using the Chao1 index, as well as the Simpson and Shannon indices, based on species-level relative abundance profiles in R. The β diversity was calculated using Bray-Curtis distance. The Linear Discriminant Analysis (LDA) Effect Size (LEfSe) method [20] was employed to compute LDA scores for the ileum microbiota. Statistical analysis using the Wilcoxon rank test and the Kruskal-Wallis H test was performed to evaluate the significant differences in the diversity index and relative abundance of taxa, KOs, KEGG pathways, and CAZy families. The results were visualized using a heatmap (*p* < 0.05). Differentially enriched KEGG pathways were identified according to reporter scores [21], with an absolute score threshold of 1.65 used to determine statistical significance.

### 2.5. Statistical Analysis

Statistical analyses were performed using the IBM SPSS Statistics program (version 21). Based on the abundance profiles, the features with significantly differential abundances across groups were identified using the Wilcoxon rank-sum test and the Kruskal-Wallis H test [22], with the Benjamini-Hochberg post hoc correction [23] applied. Pairwise comparisons were performed using the Wilcoxon signed-rank test. *p* < 0.05 was considered statistically significant, and *p* < 0.01 indicated highly significant differences.

## 3. Results

### 3.1. Metagenomic Sequencing Quality and Data Overview

In this study, metagenomic sequencing was performed on the ileal contents of six BALB/c mice, comprising three *T. gondii*-infected groups (BCA1-BCA3) and three uninfected controls (BCN1-BCN3). Paired-end sequencing (PE150) was performed using the DNBSeq platform. On average, approximately 104.36 million raw reads were generated per sample, equivalent to 15.70 GB of raw sequencing data. After quality filtering by fastp, an average of 100.26 million high-quality clean reads were retained, corresponding to a retention rate of 95.43%. The average Q20 and Q30 values were 98.08% and 94.53%, respectively, indicating high sequencing quality. The average GC content was 44.13%, with minimal variation among samples (range: 42.65–48.68%). To remove host-derived sequences, clean read segments were aligned to the mouse reference genome (GRCm39) using Bowtie2. On average, 89.46% of the clean reads were retained as non-host (microbial) sequences. The host contamination rate in the BCA group was consistently lower than that observed in the BCN group, with the lowest rate recorded in the BCA2 subgroup (3.35%). In contrast, the average host contamination rate in the BCN group was approximately 12.11% (Table S1). Overall, these results indicate that the metagenomic sequencing data obtained in this study were of high quality, with good consistency among samples, and are very suitable for subsequent studies on microbial community structure and function analysis. These high-quality metagenomic data were subsequently used for de novo assembly, microbial taxonomy analysis, functional annotation, and subsequent comparative analysis.

### 3.2. T. gondii Infection Leads to Changes in Gut Microbial Diversity in Mice

**Figure 1:**
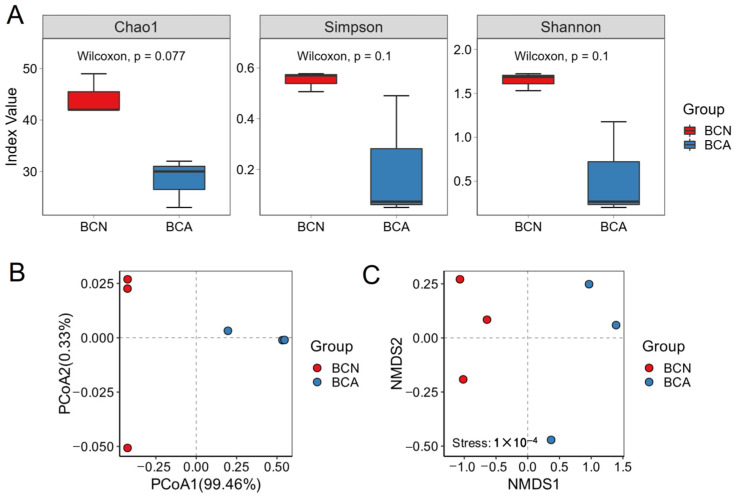
Diversity and composition of the ileal microbiota in mice. (**A**) Alpha diversity indices, including Chao1, Shannon, and Simpson indices, were used to assess microbial diversity within groups. (**B**) Principal coordinate analysis (PCoA) based on Bray–Curtis distances reveals distinct clustering between the BCA (acutely infected) and BCN (control) groups. (**C**) A non-metric multidimensional scaling (NMDS) plot based on Bray–Curtis distances further demonstrates separation between groups (PERMANOVA, *p* < 0.001). Statistical significance was evaluated using the Wilcoxon rank-sum test. BCN: uninfected control group; BCA: acutely infected group.

The α diversity represents species richness and diversity. To assess the diversity of the gut microbiota in mice, we aligned the clean read sequences with the genomic catalogue, quantified and depicted the relative abundance of bacterial species at the phylum, family, and genus levels. Overall, the gut bacterial diversity of mice was reduced after *T. gondii* infection (Figure 1A). Specifically, the Chao1, Simpson, and Shannon indices of the acute infected group (BCA) were lower than those of the control group (BCN), indicating that acute infection reduced the diversity of gut microorganisms. The Wilcoxon rank sum test revealed no statistically significant differences in the Chao1, Simpson, and Shannon indices between the groups (*p* > 0.05).

For β diversity analysis, a Bray-Curtis distance dissimilarity matrix was constructed, and principal coordinates analysis (PCoA) was used to visualize changes in microbial community composition (Figure 1B). PC1 and PC2 accounted for 99.46% and 0.33% of the total variance, respectively. PCoA showed clear separation of microbial communities between different groups. The BCA group formed a distinct cluster on the right side of the graph, clearly separated from the BCN group. NMDS further confirmed that *T. gondii* infection induced a significant alteration in the overall structure of the gut microbiota, with the BCA group and the BCN group clearly separated along the NMDS1 axis (Figure 1C, PERMANOVA, *p *< 0.001). These findings suggest that the significant clinical symptoms observed during acute *T. gondii* infection may be partly due to disturbances in the gut microbiota, with changes in the microbial community being more pronounced during the acute stage than during the uninfected stage.

### 3.3. Disturbance of Gut Microbiome Composition in Diseased Mice

**Figure 2:**
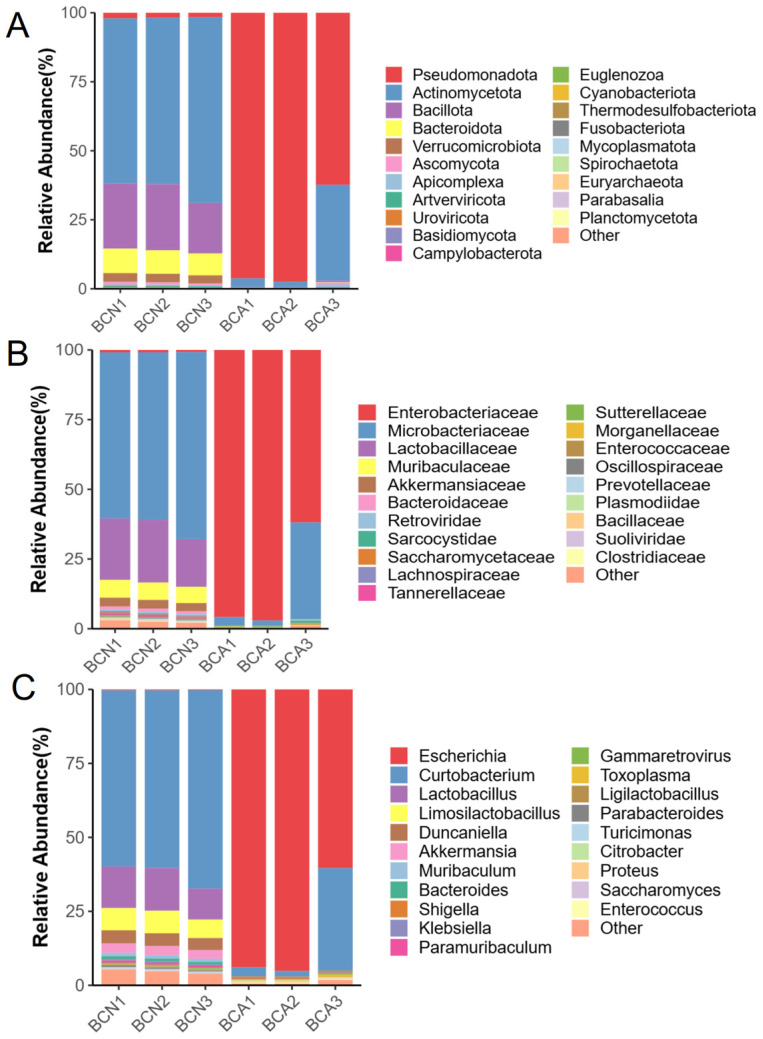
Comparison of the relative abundance of the top 20 ileal microbial taxa between groups before and after *T. gondii* infection. Relative abundance at the phylum (**A**), family (**B**), and genus (**C**) levels in the control (BCN) and acutely infected (BCA) groups. BCN: uninfected control group; BCA: acutely infected group.

Based on metagenomic data, we systematically compared the composition of gut microbial communities in mice from the BCN and BCA groups at the phylum, family, and genus levels (Figure 2A–C, Table S2). Figure 2A–C show the relative abundance of the phylum, family, and genus levels for each group of overall microbial communities. At the phylum level, the gut microbiota of the control mice (BCN) was dominated by Actinomycetota (62.46%), followed by Bacillota (21.97%) and Bacteroidota (8.43%). The proportions of these phyla decreased significantly in the infected group; in contrast, the Pseudomonadota increased markedly by 83.35% 10 days after infection.

When comparing the microbial communities at the family and genus levels, the control group (BCN) was predominantly composed of Micrococcaceae (62.26%), Lactobacillaceae (20.52%), and Muribaculaceae (6.16%), followed by Akkermansiaceae (3.08%) and Enterobacteriaceae (0.83%). In contrast, 10 days after infection with *T. gondii* in mice, the abundance of *Escherichia* species within the Enterobacteriaceae family increased significantly, reaching 84.9%. The relative abundance of *Curtobacterium* from the Microbacteriaceae family dropped to 13.26%, and the relative abundance of *Lactobacillus* from the *Lactobacillaceae* family dropped to 0.04%. At the genus level, the abundance of *Shigella* and *Escherichia* increased significantly in the BCA group. These results suggest that acute infection with *T. gondii* induces notable alterations in the ileal microbial community structure of BALB/c mice.

**Figure 3:**
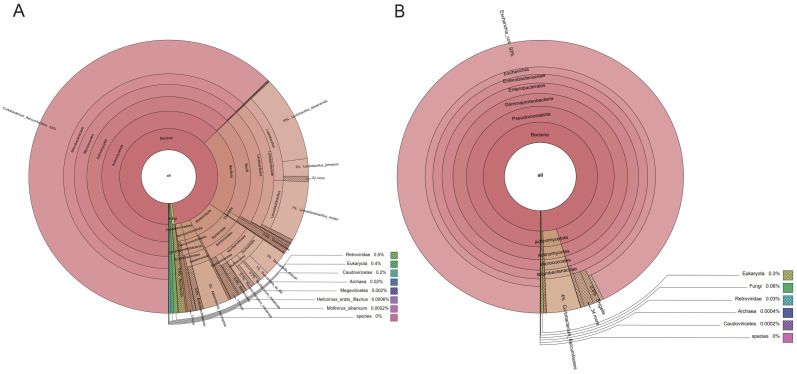
Krona plots showing the taxonomic composition of ileal microbiota in representative samples from each group after *T. gondii* infection. (**A**) The control group (BCN) exhibited a diverse and balanced community dominated by Bacteroidota, Actinobacteriota, and Bacillota, with multiple symbiotic genera present. (**B**) In contrast, the infected group (BCA) displayed a reduced diversity, with *E. coli* emerging as the predominant species.

To more intuitively illustrate the composition of the gut microbiota, we constructed a Krona plot for representative samples from each experimental group based on metagenomic sequencing data, thereby visualizing the relative abundance of taxonomic units across all taxonomic levels. The microbiota of the control group exhibited highly diverse taxonomic characteristics, including Bacteroidota and Actinomycetota, as well as various commensal bacterial genera (Figure 3A), with a stable and diverse community structure. In contrast, the microbiota structure of the infected group was highly simplified, with *E. coli* accounting for the vast majority of reads (Figure 3B). This marked difference reflects the presence of ileal microbial disturbances caused by infection and overgrowth of opportunistic bacteria.

**Figure 4:**
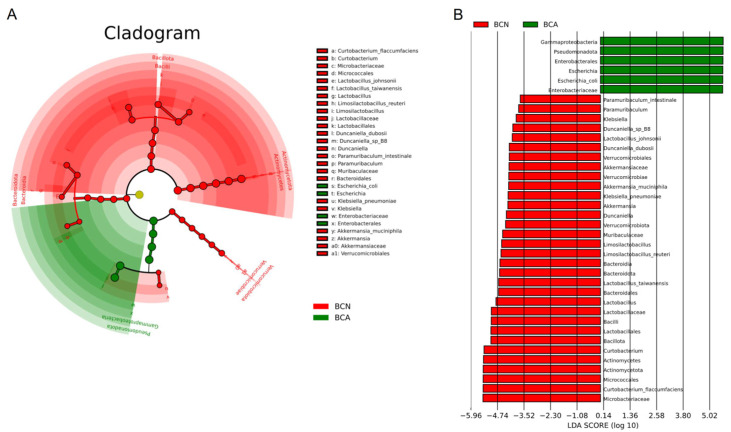
LEfSe analysis showing taxonomic differences in gut microbiota between control (BCN) and acute-infected (BCA) mice. (**A**) Cladogram highlighting taxa enriched in the BCN group (red) and the BCA group (green). (**B**) Linear discriminant analysis (LDA) scores of the differential taxa between groups.

The phylogenetic tree based on LEfSe visualized the significantly enriched taxa in the BCN and BCA groups (Figure 4, Table S3). There was a significant difference in taxonomic composition between the two groups (*p *< 0.05). The control group (BCN) was predominantly composed of Lactobacillaceae, Muribaculaceae, and Prevotellaceae, as well as several common commensal genera belonging to the phylum Actinobacteria. In contrast, the BCA group exhibited significant enrichment in various Gram-negative opportunistic pathogens, primarily within the family Enterobacteriaceae, including the genera *Escherichia* and *Shigella*, as well as other conditionally pathogenic genera classified under the phylum Pseudomonadota.

### 3.4. Functional Changes in the Gut Microbiome of Infected Mice

To explore the effects of acute *T. gondii* infection on the functional potential of mouse ileal microorganisms, we functionally annotated the metagenomic data using the KEGG database and identified a total of 4766 KO members, corresponding to 28 major metabolic and regulatory pathways in 6 samples (Table S4). These pathways mainly involve nutrient metabolism, signal transduction, immune-related mechanisms, and microbial environmental adaptation. The first-level functional classification in KEGG indicates that the primary functions of the gut microbiota are predominantly associated with the Metabolism pathway, representing 76.55%. Other notable categories include environmental information processing (9.48%), genetic information processing (6.30%), cellular processes (5.15%), human diseases (2.24%), and organism systems (0.27%). In the secondary KEGG classification, the global and overview maps (41.33%) of the overall metabolic pathways still dominated, followed by carbohydrate metabolism (11.53%), membrane transport (6.38%), amino acid metabolism (4.85%), and energy metabolism (4.09%) (Table S5).

**Figure 5:**
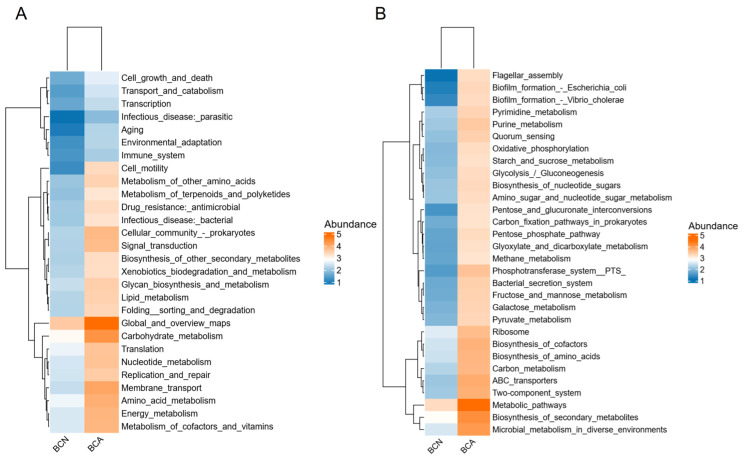
Functional differences in gut microbiota between *T. gondii*-infected (BCA) and control (BCN) mice based on KEGG annotation. (**A**) A heatmap of KEGG level 2 pathways reveals differences in the overall functional abundance of gut microbiota between the control group (BCN) and the infected group (BCA). (**B**) A heatmap of KEGG level 3 pathways highlights enhanced microbial functions in the BCA group, including core metabolism (e.g., glycolysis, pentose phosphate pathway, and amino acid metabolism), signaling systems (e.g., two-component systems and bacterial secretion systems), and biofilm formation.

We further screened the 30 most abundant secondary and tertiary KEGG pathways and drew heatmaps (Figure 5, Table S6) to evaluate the systematic differences between BCA and BCN at the microbial functional level. As shown in the heatmap, several metabolic pathways are significantly enriched in the BCA group, including glycolysis/gluconeogenesis, the pentose phosphate pathway, amino sugar and nucleotide sugar metabolism, microbial metabolism in diverse environments, and ABC transporters. At the same time, it was observed that microbial-related signaling systems, such as flagellar assembly, bacterial secretion systems, and biofilm formation in *E. coli*, were upregulated in the infected group, suggesting that *T. gondii* infection may induce functional reconstruction by regulating the metabolic capacity of the bacterial flora.

**Figure 6:**
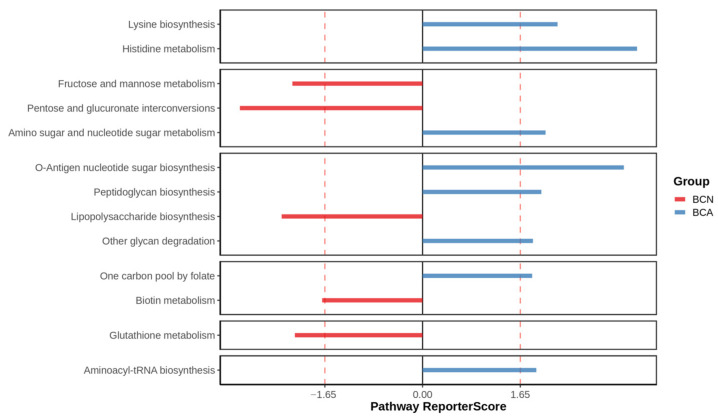
Functional differences in key metabolic pathways of mouse gut microbiota before and after *T. gondii* infection. The bar plot displays the functional shifts in 13 major KEGG pathways, with red bars indicating the BCN group and blue bars for the BCA group. A Reporter Score > 1.65 or <−1.65 indicates significant upregulation or downregulation of the corresponding pathway.

Based on metagenomic sequencing data, reporter score analysis was performed to compare the differences in key functional metabolic pathways between the control group (BCN) and the infected group (BCA; Figure 6). The results showed that the two groups exhibited significantly different functional enrichments in multiple important pathways, suggesting that the infection induced gut microbiota functional profile reconstruction. Significant upregulation of the histidine metabolism and O-Antigen nucleotide sugar biosynthesis pathways was observed in the BCA group. Meanwhile, amino sugar and nucleotide sugar metabolism, as well as phenylalanine, tyrosine, and tryptophan biosynthesis, were also active. In contrast, the BCN group showed significantly suppressed fructose and mannose metabolism, pentose and glucuronate interconversions, lipopolysaccharide (LPS) biosynthesis, bioin metabolism, and glutathione metabolism.

**Figure 7:**
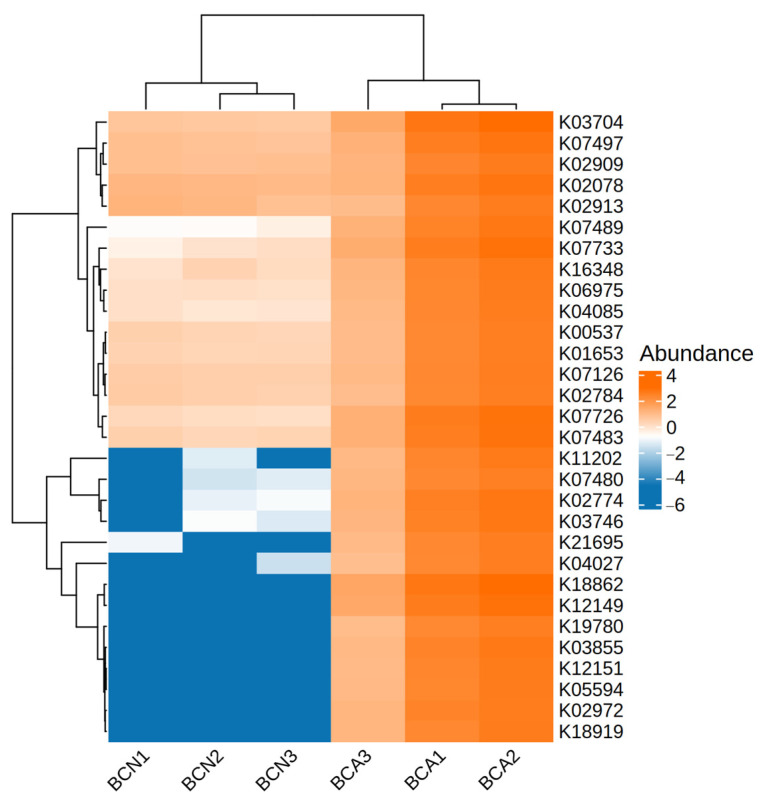
Heatmap of differentially abundant KEGG Orthologs (KOs) in gut microbiota between control (BCN) and acute-infected (BCA) mice. The heatmap shows normalized abundance levels of selected KO functional genes across samples. Orange indicates higher abundance, while blue represents lower expression levels. Multiple KO entries, such as K02784, K02078, and K02774, were significantly enriched in the BCA group.

To further verify the effect of acute *T. gondii* infection on gut microbiota functions and composition, we conducted a cluster analysis of the abundance of functional genes in the gut microbiota of mice in the BCN and BCA groups based on KEGG Orthologs (Figure 7, Table S7). The heatmap shows the differential expression trends of multiple KO pathways closely related to bacterial structure and function in the two groups of samples. The results showed a series of significantly upregulated functional genes in the BCA group, such as K02784, K02078, K02774, and K04085. In contrast, the expression levels of these KO pathways were significantly reduced in the BCN group, and some pathways were even missing, suggesting that their microecosystems are more inclined to a stable symbiotic environment.

**Figure 8:**
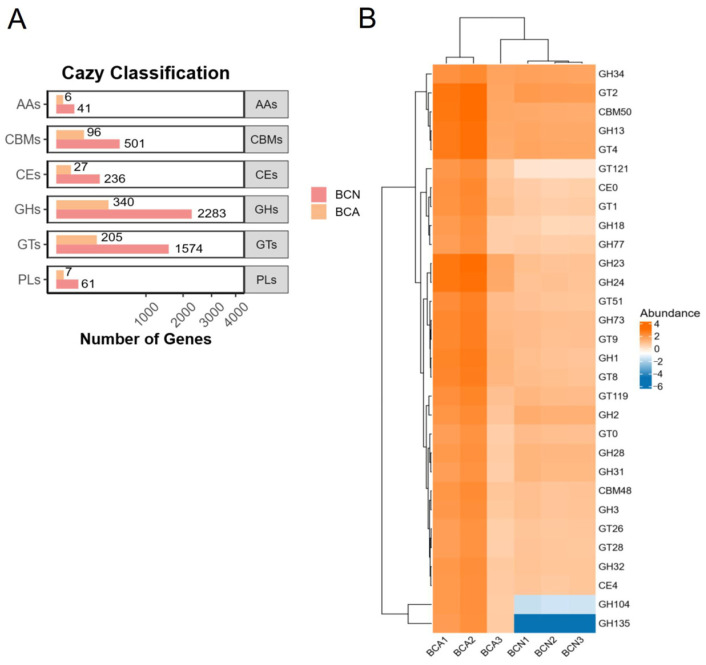
CAZy classification and abundance comparison of gut microbiota carbohydrate-active enzymes between control (BCN) and acute-infected (BCA) mice. (**A**) Bar graph showing that the number of genes annotated to six CAZy enzyme categories (GHs, GTs, CBMs, CEs, PLs, AAs) is significantly reduced in the BCA group compared to the BCN group, with the most marked decline in glycoside hydrolases (GHs) and glycosyltransferases (GTs). (**B**) Heatmap showing the abundance distribution of main CAZy families in different samples, with significant upregulations of certain families (e.g., GH23, GH24, GT4, and GT2) in the infected group.

To further analyze the effect of acute *T. gondii* infection on the carbohydrate metabolic potential of host gut microbiota, an annotation analysis was performed for the BCA and BCN groups on the Carbohydrate-Active enZymes (CAZy) database. As shown in Figure 8A, the number of annotated genes across all six classes of enzymes (GHs, GTs, CBMs, CEs, PLs, and AAs) was significantly higher in the BCN group than in the BCA group. Among them, the difference in glycoside hydrolases (GHs) was the most significant (BCN: 2283 vs. BCA: 340). Similar trends were also observed in glycosyltransferases (GTs; BCN: 1574 vs. BCA: 205), carbohydrate binding modules (CBMs; 501 vs. 96), carboxylesterases (CEs; 236 vs. 27), polysaccharide lyases (PLs; 61 vs. 7), and auxiliary activity enzymes (AAs; 41 vs. 6). In addition, we identified functional pathways that differed significantly between groups before and after *T. gondii* infection. At the enzyme family level, it manifested as upregulation of glycoside hydrolases (GHs) and glycosyltransferases (GTs), including the significantly upregulated expression in several CAZy families like GH23, GH24, GT4, and GT2 (Figure 8B, Table S8), indicating that *T. gondii* infection triggered marked restructuring of the CAZy enzyme profile of the gut microbiota.

## 4. Discussion

Oral ingestion of *T. gondii* cysts or oocysts constitutes the primary transmission route for toxoplasmosis in humans and animals [24]. Murine models of oral infection consistently demonstrate profound disruption of gut homeostasis, with disease progression closely linked to gut microbiota dynamics [25]. Recent reviews synthesize evidence that acute-phase *T. gondii* infection remodels gut microbiome composition, notably through decreased Bacillota abundance and increased Bacteroidota [26,27]. While these taxonomic shifts are increasingly documented, the molecular mechanisms governing functional interactions between *T. gondii* and commensal microbiota remain poorly defined. To address this gap, we characterized gut microbial communities in a murine infection model at peak acute phase (day 10 post-infection), with particular focus on functional metagenomic alterations and associated metabolic pathway dysregulation.

Our results clearly showed that acute *T. gondii* infection significantly disrupts the gut microbiota composition and function of the host. On day 10 post-infection, the gut microbiota of infected mice showed significantly decreased α diversity (Figure 1A), suggesting reduced microecosystem complexity and stability. PCoA analysis further confirmed that *T. gondii* infection significantly altered the overall microbial community structure (Figure 1B), consistent with previous findings that parasitic infection induces microbiota remodeling [6,28,29]. In terms of taxonomic structure, the proportion of common commensal bacteria, such as Actinobacteriota, Bacillota, and Bacteroidota, in the infected group was significantly reduced compared to the uninfected control group. In contrast, potential opportunistic pathogens, such as Pseudomonadota, increased greatly, resulting in a severe imbalance in the gut microbiota structure (Figure 2A). Evidence suggests that the relative abundance of Pseudomonadota is related to the ecological imbalance of the gut microbial community in the host with enteritis, making it a potential diagnostic marker [30].

Notably, this study highlights the sensitivity of Actinobacteriota in *T. gondii* infection. Although previous research mostly focused on the changes in Bacillota, Bacteroidota, and Pseudomonadota after infection [7,11], This study found a significant decrease in Actinobacteriota in the infected group (Figure 2A), suggesting their important role in maintaining gut homeostasis and resisting pathogen invasion, aligned with the results of recent studies focusing on their function in maintaining the gut barrier [31]. Genus-level analysis showed the significant enrichment of multiple opportunistic pathogens under Pseudomonadota in the gut of infected mice. Metagenomic data showed significantly amplified Enterobacteriaceae bacteria in the infected group, especially *Escherichia* (83.14%), *Shigella* (0.73%), *Citrobacter* (0.35%), *Proteus* (0.33%), *Salmonella* (0.27%), and a small amount of *Pseudomonas* (0.01%) (Figure 2B,C). These bacteria typically possess a strong ability to induce inflammation and compromise the mucosal barrier, and their proliferation is often closely linked to microecological dysbiosis and inflammation [32,33].

This study found that the structural changes in microbiota caused by *T. gondii* infection largely overlap with the characteristics of inflammatory bowel disease (IBD). On the one hand, the rapid *E. coli* amplification during *T. gondii* infection and the significant upregulation of LPS-related biosynthetic pathways are known landmark changes in IBD [34]. Multiple studies have reported a decreased ratio of Actinobacteria to Bacteroidetes [35,36,37,38,39] and the abnormal expansion of Enterobacteriaceae [40,41] in IBD patients, which is similar to the microbiota structure changes of the infected group in this study. In addition, LPS can induce the release of pro-inflammatory cytokines such as TNF-α and IL-6 by activating the TLR4 signaling pathway, which destroys the gut epithelial barrier and increases gut permeability, thereby forming a positive inflammatory feedback loop [34,42]. These mechanisms suggest that, in addition to causing gut microbiota imbalance, *T. gondii* infection also creates a pathological environment similar to IBD through abnormal activation of metabolic pathways. Furthermore, this study found that the ratio of Bacillota to Bacteroidota decreased significantly in the infected group, and this indicator has been widely used to assess gut inflammatory status, especially in IBD and irritable bowel syndrome [43,44]. In addition, Su et al. found that removing the gut commensal microbiota prolonged the survival time of mice infected with *T. gondii*, whereas the pathological exacerbation of interferon-γ (IFN-γ) on *T. gondii* induced intestinal pathology depends on the presence of commensal microbiota, further supporting the synergistic pathogenesis among *T. gondii*, microbiota, and immune system [45]. Based on the above findings, we observed significant enrichment of Enterobacteriaceae in the infected mice (Figure 2, Figure 3 and Figure 4). The high abundance of pathogenic *E. coli* is consistent with species composition results, which may lead to gut inflammation and dysfunction after *T. gondii* infection [46].

In terms of functional analysis, the KEGG annotation results showed the enrichment of a large number of metabolic pathways supporting pathogen growth in the infected group, such as the significant upregulation of metabolic pathways, bacterial secretion system, biofilm formation of *E. coli*, and microbial metabolism in diverse environments (Figure 5), suggesting that *T. gondii* infection induces broad functional shifts of microbiota functions and metabolism. The Reporter Score analysis results, particularly the significantly upregulated O-Antigen nucleotide sugar biosynthesis pathway, are consistent with the abnormal amplification of Gram-negative bacteria such as *E. coli* (Figure 6). As the outermost variable structure domain of LPS, O-antigen consists of repeated oligosaccharide units [47], and its synthesis is one of the key steps in LPS synthesis [48,49]. LPS is a potent pro-inflammatory microbial product that stimulates cytokine cascade reactions and caspase activation mediated by Toll-like receptor 4 (TLR4) [50]. In addition to causing local gut inflammation in the host, LPS also crosses the damaged barrier into the circulation, where it interacts with cytokines, leading to systemic inflammation [39,42,51]. The clinical manifestation of gut inflammation after *T. gondii* infection may be partially attributed to the genes of the large number of LPS that bind to the inflammasome sensors. This study found relatively higher expression levels of multiple KOs, such as K02078 and K02774, in the infected group (BCA), which are genes widely involved in cell wall assembly, LPS synthesis, membrane protein transport, and other bacterial processes (Figure 7). In contrast, the abundance of these genes was generally low in the control group (BCN), and some pathways were even completely missing, suggesting that the microecosystem trends toward stable commensalism and a low inflammatory environment. K02078 encodes LpxC (a deacetylase), which catalyzes the first step of LPS synthesis (UDP-3-O-(R-3-hydroxyacyl)-N-acetylglucosamine deacetylase) [52] and the speed-limiting step of LPS synthesis [53,54]. Lipid A is the hydrophobic core of LPS and is critical to its structure and function. On the other hand, K02774 encodes WaaG, a glycosyltransferase involved in the synthesis of LPS core oligosaccharides [55] and catalyzes the addition of glucose to lipid A core oligosaccharides by UDP-glucose, and its structural diversity affects the immunogenicity of LPS and the pathogenicity of bacteria [56].

In addition, this study also showed enhanced synthetic pathways of phenylalanine, tyrosine, and tryptophan in the acute *T. gondii* infection group (Figure 6). *T. gondii* infection limits the availability of tryptophan in the host since it is an essential amino acid of *T. gondii* [57]. During infection, IFN-γ stimulates the release of indoleamine-pyrrole 2,3-dioxygenase (IDO) from the host cell, resulting in increased tryptophan decomposition [58]. The tryptophan catabolites kynurenic acid and quinolinic acid increase oxidative stress in the brain, destroy cells, and eventually lead to apoptosis [59]. Tryptophan is also a precursor in the synthesis of neurotransmitter serotonin, and its metabolic changes may reflect the utilization of host amino acids by *T. gondii* or the neurotransmitter metabolism disorders during acute infection. On the other hand, the tryptophan metabolism of gram-negative microbes may produce metabolites that affect pathological and immune responses through the AhR signaling pathway [60]. Dos Santos et al. demonstrated that antibiotic-induced gut microbiota depletion enhances resistance to *T. gondii* infection, and this depletion protects mice from ileitis caused by *T. gondii* [61]. The depletion of gram-negative bacteria results in systemic elevation of tryptophan levels but does not affect the activity of IDO in mice, thereby enhancing defense against infection. The depletion of gram-positive bacteria has no such effect. These findings suggest that the gut microbiota regulates host-microbiota interactions during *T. gondii* infection by regulating the availability of tryptophan.

Several limitations of this study warrant consideration. Firstly, the findings are derived from a murine model of acute oral infection. While this model is valuable for mechanistic insights, the epidemiology, disease progression, and host immune responses to *T. gondii* differ between mice and humans, limiting direct extrapolation of results. Secondly, the small sample size (*n* = 3 per group) reduces statistical power and may constrain the generalizability of some observations. Future studies with larger cohorts are needed to confirm these findings. Thirdly, our analysis focused exclusively on the acute phase (day 10 post-infection). The dynamics of gut microbiota dysbiosis and functional alterations during the chronic phase of infection, which is highly relevant to human toxoplasmosis, remain unexplored.

To sum up, we speculate that the metabolic landscape shaped by *T. gondii* infection involves a precise balance between the parasites’ nutritional resource “competition” with the host and the microbiota and the “defense” of the host and the microbiota to maintain homeostasis. The observed microbiota metabolic alterations may support *T. gondii* replication and infection establishment by potentially providing substances and energy, while also potentially triggering host defensive responses. Dysregulated gut microbiota may play dual roles in this process. As a metabolic hub, it may nourish the parasite while indirectly participating in host immune regulation. Understanding the pathways of these specific perturbations is crucial for elucidating the pathogenesis of *T. gondii.* For example, controlling amino acid and energy intake by regulating diet or colonizing beneficial bacteria may provide a strategy for influencing host metabolism, thereby preventing or treating toxoplasmosis. In the case of the CAZy family, studies have shown that the glycosyltransferase families GT2 and GT4 enriched in infected mice are involved in LPS biosynthesis, playing a key role in the synthesis of O-antigen polysaccharides, which appear to be the cause of gut inflammation [49,62].

Collectively, this study provides a detailed characterization of acute gut microbiota dysbiosis and functional reprogramming in a murine model of *T. gondii* infection. We identified specific taxonomic shifts (e.g., depletion of Actinobacteriota, enrichment of Enterobacteriaceae) and functional alterations (e.g., upregulation of O-antigen biosynthesis, tryptophan pathways) linked to inflammation and barrier disruption. While acknowledging the limitations of the murine model and acute phase focus, these findings offer mechanistic insights into host-microbiota-parasite interactions. The parallels observed between infection-induced dysbiosis and IBD-like features suggest potential shared pathogenic mechanisms involving microbial metabolites (e.g., LPS) and immune activation. Further investigation in human cohorts and chronic infection models is essential to determine the translational relevance of these microbial and metabolic signatures for understanding toxoplasmosis pathogenesis.

## 5. Conclusions

In summary, this study systematically characterizes acute *T. gondii* infection-induced gut microbiota dysbiosis in mice, revealing significant microbial ecosystem destabilization through reduced microbial diversity, community restructuring, and impaired metabolic functions. Critically, infection promoted the proliferation of opportunistic pathogens such as Pseudomonas, accompanied by upregulation of metabolic pathways including O-antigen nucleotide sugar biosynthesis and tryptophan biosynthesis. These alterations suggest potential mechanisms through which microbiota dysbiosis may exacerbate host inflammation and disease progression, potentially involving immune activation, gut barrier disruption, and metabolic imbalance. Collectively, our findings advance the understanding of host–microbiota interactions during *T. gondii* infection and highlight the critical role of microbiota dysbiosis in mediating intestinal inflammation and pathology. This work provides foundational insights into the dynamic regulatory mechanisms of the “gut microbiota–immunity–*T. gondii*” axis, which may inform future investigations into preventive and therapeutic approaches.

## Data Availability

The metagenomic sequencing data reported in this research have been deposited in the China National Center for Bioinformation (https://www.cncb.ac.cn/, accessed on 12 July 2025, accession number: PRJCA042718).

## Supplementary Materials

The following supporting information can be downloaded at: https://www.mdpi.com/article/10.3390/microorganisms13092056/s1, Table S1: Summary of metagenomic sequencing quality metrics; Table S2: Differential abundance analysis of the top 30 microbiota between BNA and BCN groups at the phylum, family, genus levels; Table S3: LEfSe analysis of differential microbiota between BCA and BCN groups during the recovery period; Table S4: Predicted KEGG Orthologs (KOs) and their relative abundances in the BCA and BCN groups; Table S5: Number of microbial genes mapped to KEGG pathways at levels 1 and 2 in the BCA and BCN groups; Table S6: KEGG pathways with significant differences at level 2 and 3 between the BCA and BCN groups; Table S7: Cluster analysis of KO-based functional gene abundance in the intestinal microbiota of the BCA and BCN groups; Table S8: CAZy enzyme families with significantly different functional pathway abundances between the BCA and BCN groups.

## Abbreviations

The following abbreviations are used in this manuscript:

DNBSeq	DNA Nanoball Sequencing
PE150	Paired-End 150 bp Sequencing
KEGG	Kyoto Encyclopedia of Genes and Genomes
CAZy	Carbohydrate-Active enZymes
KO	KEGG Ortholog
LDA	Linear Discriminant Analysis
LEfSe	Linear Discriminant Analysis Effect Size
PCoA	Principal Coordinates Analysis
NMDS	Non-metric Multidimensional Scaling
PERMANOVA	Permutational Multivariate Analysis of Variance
LPS	Lipopolysaccharide
TLR4	Toll-Like Receptor 4
IFN-γ	Interferon gamma
IDO	Indoleamine 2,3-dioxygenase
AhR	Aryl Hydrocarbon Receptor
GHs	Glycoside Hydrolases
GTs	GlycosylTransferases
CBMs	Carbohydrate-Binding Modules
CEs	Carboxylesterases
PLs	Polysaccharide Lyases
AAs	Auxiliary Activityies
